# Construction and Quality Analysis of Transgenic *Rehmannia glutinosa* Containing TMV and CMV Coat Protein

**DOI:** 10.3390/molecules21091134

**Published:** 2016-08-27

**Authors:** Zhongqiu Teng, Ye Shen, Jing Li, Zhongping Lin, Min Chen, Min Wang, Man Li, Hongran Dong, Luqi Huang

**Affiliations:** 1 State Key Laboratory Breeding Base of Dao-Di Herbs, National Resource Center for Chinese Materia Medica, China Academy of Chinese Medical Sciences, Beijing 100700, China; zhongqiuteng@163.com (Z.T.); shenye70@hotmail.com (Y.S.); justinworking@126.com (J.L.); cm315keke@163.com (M.C.); wangmincacms@sohu.com (M.W.); liman887@163.com (M.L.); dhyopp@126.com (H.D.); 2 State Key Laboratory for Infectious Disease Prevention and Control, National Institute for Communicable Disease Control and Prevention, Chinese Center for Disease Control and Prevention, Beijing 102206, China; 3 Collaborative Innovation Center for Diagnosis and Treatment of Infectious Diseases, Hangzhou 310003, China; 4 National Key Laboratory of Protein Engineering and Plant Genetic Engineering, College of life science, Peking University, Beijing 100871, China; linzp@pku.edu.cn

**Keywords:** virus resistant, *Rehmannia glutinosa*, transgenic plant, medical quality, biosafety, TMV & CMV

## Abstract

Plant viruses, especially tobacco mosaic virus (TMV) and cucumber mosaic virus (CMV) are serious threats to *Rehmannia glutinosa* which is a “top grade” herb in China. In the present study, TMV- and CMV-resistant *Rehmannia glutinosa* Libosch. plants were constructed by transforming the protein (CP) genes of TMV and CMV into *Rehmannia glutinosa* via a modified procedure of *Agrobacterium tumefaciens*-mediated transformation. Integration and expression of TMV CP and CMV CP transgenes in 2 lines, LBA-1 and LBA-2, were confirmed by PCR, Southern blot and RT-PCR. Both LBA-1 and LBA-2 were resistant to infection of homologous TMV and CMV strains. The quality of transgenic Rehmanniae Radix was evaluated based on fingerprint analysis and components quantitative analysis comparing with control root tubes. These results showed that chemical composition of transgenic Rehmanniae Radix were similar to non-transgenic ones, which demonstrated that the medical quality and biosafety of transgenic Rehmanniae Radix were equivalent to non-transgenic material when consumed as traditional Chinese medicinal (TCM).

## 1. Introduction

*Rehmannia glutinosa* Libosch. (Dihuang), a comprehensive traditional Chinese medicinal (TCM) herb, was considered as a “top grade” herb in China [1]. Raw and processed Rehmanniae Radix are reported to possess many kinds of pharmacological activity, such as clearing away heat [2], promoting salivation [3], nourishing “yin” [4], promoting the production of body fluids [5] and benefitting the marrow [6], etc. Supply of the medical material is becoming increasingly scarce due to production reductions and increasing quality requirements. Germplasm degradation caused by tobacco mosaic virus (TMV) and cucumber mosaic virus (CMV) infection is one of important factors which has a huge impact on the production of *Rehmannia glutinosa* [7,8]. A coat protein-mediated resistance (CP-MR) strategy against plant viruses has been established and applied in the development of transgenic plants [9]. Powell-Abel et al. reported that transgenic tobacco plants with the coat protein genes of TMV showed delayed appearance of severe TMV symptoms [10]. After decades of development, virus-resistant transgenic plants producing certain CPs of different viruses, including TMV [11], CMV [12], soybean dwarf virus (SbDV) [13], alstroemeria mosaic virus (AIMV) [14], potato virus X (PVX) [15], potato virus Y (PVY) [16] and so on, were established. Hence, transgenic CP-MR strategy offers a powerful tool to overcome plant virus diseases. The development of *Rehmannia glutinosa* cultivars resistant to TMV and CMV is necessary because of the serious damage to its production.

In the present work, we describe a procedure for transformation of *Rehmannia glutinosa* Libosch. (cultivar “Wen 85-5”). We transformed a construct containing the CP genes of TMV and CMV into wild *Rehmannia glutinosa* to acquire TMV and CMV resistant *Rehmannia glutinosa* plants. The expression of transgenes in transgenic progenies was confirmed. TMV and CMV resistance were also investigated. More important, the trial here involved construction of a kind of transgenic traditional Chinese medicine which is frequently used in the clinic. The medicinal quality and biosafety of transgenic Rehmanniae Radix was assessed via comparison of UPLC-MS fingerprints, the content of polysaccharide and active components with the wild counterpart.

## 2. Results

### 2.1. Establishment and Optimization of Transformation System

The morphogenetic ability of different varieties of *Rehmannia glutinosa* is different due to the distinct genotypes. Experimental conditions such as hormone type and concentration, illumination time and environmental temperature, etc. have a significant effect on the time of callus induction and bud differentiation. In the present study, an L16 (45) orthogonal array was used to optimize of transformation conditions. All five variables (pre-culture time, co-culture time, infection time, infection concentration and phytohormones) were varied at four levels (Supplementary Table S1). The results (Supplementary Tables S2–S4) indicated that pre-culture time, co-culture or the five factors of time, time of infection, bacteria concentration and hormone had no significant impact on sprouting rate. Pre-culture time concentration of bacterium and co-culture time has remarkable influence on the differentiation rate of positive plants on account of the higher F value (Supplementary Table S4). The optimum conditions for *Rehmannia glutinosa* leaf genetic transformation were as follows: *Rehmannia glutinosa* leaves free of pre-culture were inoculated with *Agrobacterium* (OD_600_ = 0.4) for 8 min; after 36 h of co-culture, transfer the leaves to differentiation medium (MS + 6-BA 2 mg/L + NAA 0.5 mg/L + cefotaxime 100 mg/L) and refresh the culture medium every 20 days until the differentiation of adventitious buds; and then transfer the adventitious bud to the rooting medium.

### 2.2. Construction of Transgenic Plants

**Figure 1:**
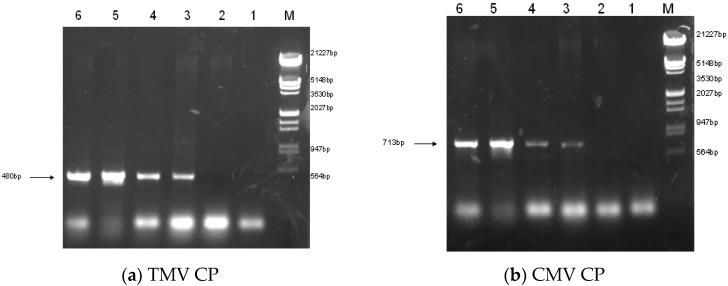
The TMV CP gene (**a**) and CMV CP gene (**b**) fragments amplified by PCR. DNA fragments with expected sizes (TMV CP gene: 480 bp; CMV CP gene: 713 bp) were observed in LBA-1, LBA-2 and LBA-3; no PCR product appeared from wild type (85-5). M: Marker; 1: Control (ddH_2_O); 2: PCR product of 85-5; 3: PCR product of LBA-1; 4: PCR product of LBA-2; 5: PCR product of LBA-3; 6: PCR product of pCAM-TM35SCM.

**Figure 2:**
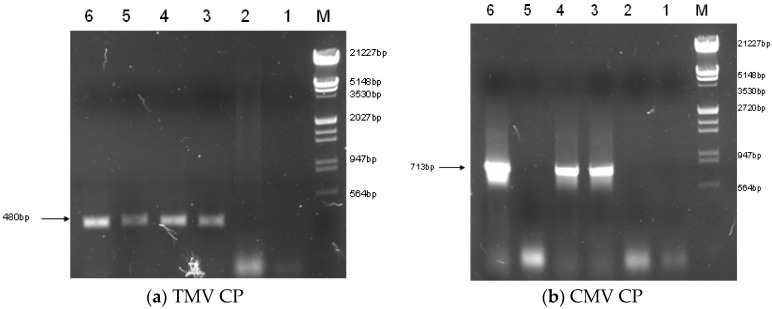
RT-PCR analysis of the TMV CP gene (**a**) and CMV CP gene (**b**). The expected size of the amplified portion of the TMV CP gene transcript were observed in LBA-1, LBA-2 and LBA-3; a fragment of ca. 713 bp, corresponding to the amplified portion of the CMV CP gene transcript were observed in LBA-1 and LBA-2, but absent in LBA-3. M: Marker; 1: Control (ddH_2_O); 2: PCR product of 85-5; 3: PCR product of LBA-1; 4: PCR product of LBA-2; 5: PCR product of LBA-3; 6: PCR product of pCAM-TM35SCM.

**Figure 3:**
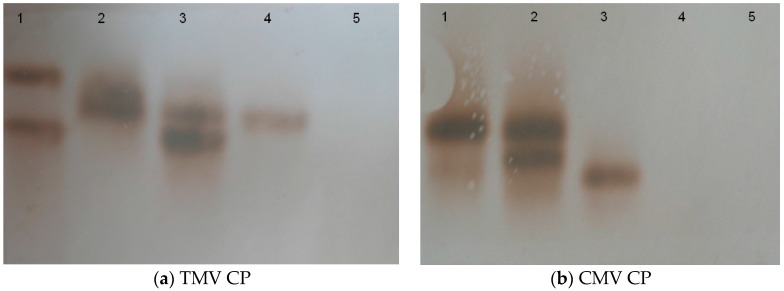
Southern analysis of transgenic plants. The Southern blot analysis demonstrated the presence of TMV CP gene (**a**) and CMV CP gene (**b**) copy in the genome of LBA-1 and LBA-2. 1: positive control; 2: LBA-1; 3: LBA-2; 4: LBA-3; 5: 85-5.

Binary vector constructions were transferred into the disarmed *Agrobacterium* strain LBA4404 and subsequently into *Rehmannia glutinosa*. Three PPT resistant plants (LBA-1, LBA-2 and LBA-3) were finally obtained from 120 of leaf-disc after infection of *Agrobacterium* containing pCAM-TM35SC plasmid. The three independent transgenic lines were identified based on PCR, RT-PCR and also by genomic Southern blot analysis. PCR showed that the three transgenic *Rehmannia glutinosa* lines contained TMV-CP and CMV-CP genes (Figure 1). RT-PCR suggested that LBA-1 and LBA-2 contained markedly enhanced amounts of TMV-CP and CMV-CP mRNA. However, LBA-3 was free of CMV-CP mRNA even though TMV-CP gene can be expressed in the transgenic line (Figure 2). We also got similar results in the genomic Southern blot analysis. Both TMV-CP gene and CMV-CP gene were confirmed to be integrated into the chromosomes of LBA-1 and LBA-2 by Southern blotting. The Southern blotting analysis of LBA-3 indicated that only TMV-CP was integrated into chromosomes of the line, but CMV-CP was not (Figure 3). Therefore, transgenic lines LBA-1 and LBA-2 were used for further studies.

### 2.3. Virus Resistance of Transgenic Lines

To assess virus resistance of transgenic lines expressing the TMV-CP and CMV-CP (LBA-1 and LBA-2), and wild type *Rehmannia glutinosa* plants were infected by biotrophic TMV and CMV.

**Figure 4:**
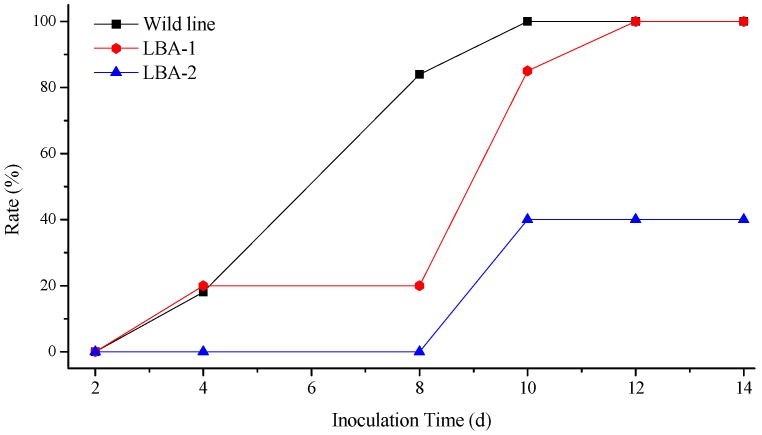
Incidence of *Rehmannia glutinosa* after inoculation of TMV.

**Figure 5:**
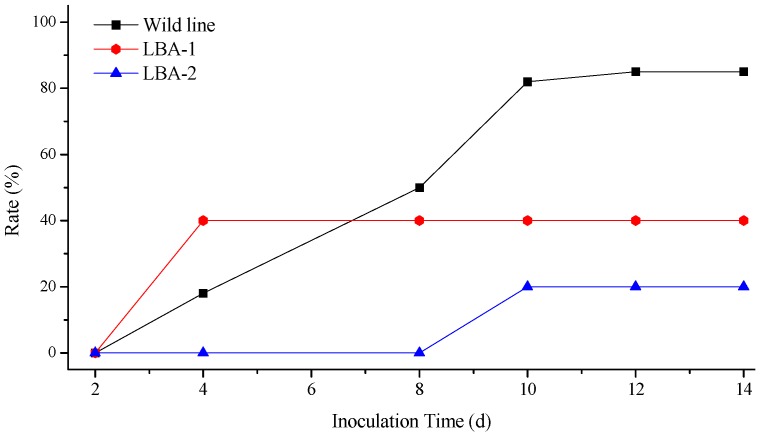
Incidence of *Rehmannia glutinosa* after inoculation of CMV.

**Figure 6:**
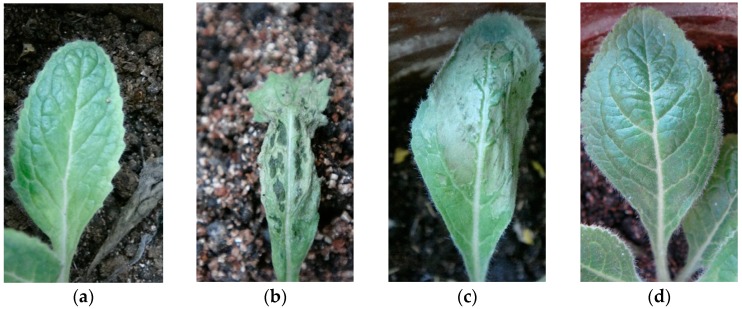
Symptoms of *Rehmannia glutinosa* leaves 10 days after inoculation of the TMV. (**a**) control; (**b**) wild line; (**c**) LBA-1; (**d**) LBA-2.

**Figure 7:**
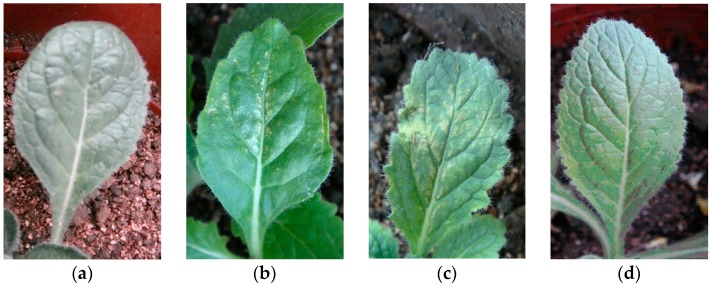
Symptoms of *Rehmannia glutinosa* leaves 10 days after inoculation with CMV. (**a**) control; (**b**) wild line; (**c**) LBA-1; (**d**) LBA-2.

The illness conditions of test plants were linked to the virus inoculation concentration. High virus concentration causes serious *Rehmannia glutinosa* illness, and even death. On the contrary, the illness maybe too light to evaluate illness conditions when the virus concentration is too low, so the best inoculation concentration of TMV and CMV was investigated before the virus resistance test (Supplementary Table S5). Finally, 0.1 μg/mL of virus was selected as the optimum concentration. Figure 4 shows the incidence of *Rehmannia glutinosa* infected by TMV. The incidence rate of LBA-1 and LBA-2 was significantly less than in the wild line within 8 days after inoculation. LBA-2 maintained a low incidence for more than 10 days. However, all the LBA-1 and wild line *Rehmannia glutinosa* plants suffered TMV disease after 8 days. The disease index test was also determined in order to investigate the TMV resistance of *Rehmannia glutinosa* according to grade and tobacco disease investigation method of (Industry criteria of the tobacco industry of China, YC/T 39-1996). The disease indexes of LBA-1, LBA-2 and wild type *Rehmannia glutinosa* were 42.9, 37 and 15, respectively. LBA-2 was thus classified as a resistant plant (5 < disease index ≤ 20), which showed excellent resistance to TMV virus. LBA-1 was a medium resistance plant (20 < disease index ≤ 40), which TMV resistance was slightly higher than that of the wild type (susceptible plant, 40 < disease index ≤ 60). The same experiment was performed on CMV resistance of *Rehmannia glutinosa*. LBA-1 showed CMV disease symptoms soon after CMV inoculation, but showed a significantly lower incidence compared with wild type *Rehmannia glutinosa* during the observation time, as shown in Figure 5. The CMV disease incidence of LBA-2 was lower than that of LBA-1 and the wild lines. In addition, LBA-2 was a resistant plant (disease index = 10), and LBA-1 was a medium resistance plant (disease index = 30). We also compared the symptoms of *Rehmannia glutinosa* leaves 10 days after inoculation with the virus (Figure 6 and Figure 7).

The virus infection symptoms of LBA1 were less than those of the wild lines. Most inoculated leaves of LBA-2 almost showed no symptoms. In summary, both LBA-1 and LBA-2 were TMV and CMV resistant *Rehmannia glutinosa* lines, and LBA-2 showed better virus resistance as measured by disease index and disease incidence tests than LBA-1.

### 2.4. Quality Evaluation of Transgenic Rehmannia glutinosa

Using the UPLC-MS fingerprint of wild Rehmanniae Radix as the standard pattern against which to compare other preparations, two different genotypes of Rehmanniae Radix samples were comparatively analyzed (Supplementary Figure S4). The similarities between the chromatograms of the two groups of samples (*n* = 10) compared with the reference fingerprint were close to 1, as shown in Table 1 and Supplementary Figure S4c. The results indicated that the chromatographic patterns of transgenic samples were consistent with the wild type Rehmanniae Radix.

Catalpol, aucubin, leonuride, acteoside, echinacoside and polysaccharides are considered the bio-active components of Rehmanniae Radix. The contents of the compounds and polysaccharides in transgenic Rehmanniae Radix compared with the wild type are shown in Table 2. The contents of the five compounds and total polysaccharides in transgenic type were closed to that of wild types. Moreover, the contents of analytes in every group of Rehmanniae Radix showed no statistically differences. The results indicated that the medical quality of transgenic Rehmanniae Radix maybe equivalent to non-transgenic material when consumed as a traditional Chinese medicine.

## 3. Discussion

TMV and CMV infections reduce agricultural productivity worldwide [17]. Combined infection of these two kinds of plant virus causes more severe symptoms, even yellow withered crops [18,19]. Breeding virus-resistant plants via gene modification is considered an effective way to control virus infection [20,21,22]. Cultivation of *Rehmannia glutinosa*—a kind of the bulk Chinese herbal medicine—suffers from infection by TMV and CMV in producing areas. Coat protein mediated protection (CPMP) has been established in transgenic plants against many kinds of plant virus [23,24,25]. The same strategy was used here to develop virus-resistant *Rehmannia glutinosa* transformed with the CMV-CP and TMV-CP genes. Two transgenic lines (LBA-1 and LBA-2) were confirmed to express both exogenous virus CP genes. In order to test the antiviral capacity of *Rehmannia glutinosa* lines, transgenic *Rehmannia glutinosa* and wild line were inoculated with TMV and CMV. Both LBA-1 and LBA-2 were TMV and CMV resistant *Rehmannia glutinosa* lines. However, the antiviral capacities of the two transgenic lines were different, as LBA-2 showed better virus resistance as measured by disease index and disease incidence tests than LBA-1.

Transgenic technology can improve certain characteristics of the medicinal plants, such as antiviral resistance, improved resistance to diseases in general and enhanced contents of effective compounds. As a commonly used Chinese herbal medicine, *Rehmannia glutinosa* is different from some other medicinal plants which often used as raw materials for extracting effective components. For example, *Artemisia carvifolia* is used for the extraction of antimalarial constituents [26]. *Rehmannia glutinosa* has always been applied in Chinese medicine as a decoction which is taken orally after decocting in water, or directly used in proprietary Chinese medicine preparations. Therefore, we need to pay attention to the quality and safety of transgenic *Rehmannia glutinosa.* In the present study, contents of several major bio-active compounds (three iridoid glycosides and two phenethyl alcohol glycosides) and total polysaccharides (considered as the main constituents responsible for the tonic effect) in transgenic *Rehmannia glutinosa* were compared with their wild counterpart. Furthermore, fingerprint technology which has been widely used in quality control of TMC [27,28] was applied in a comparative study between transgenic and wild *Rehmannia glutinosa.* The tubers of transgenic *Rehmannia glutinosa* showed similar chemical compositions as their wild counterpart. Chemical composition is the material basis of Chinese herbal medicine efficacy. The similar chemical compositions of different *Rehmannia glutinosa* suggest that exogenous gene introduction had no effect on the medicinal quality of the medical plant. Consumers and regulatory authorities pay more attention to the safety of transgenic plants. Substantial equivalence is considered as the principal criterion for assessing the safety of novel foodstuffs, the principle being that, composition of the novel (i.e., transgenic) foodstuff should not differ in a meaningful way from a traditional (i.e., unmodified) variety(s) [29,30], so the similar chemical composition of transgenic and wild *Rehmannia glutinosa* also provides preliminary evidence of the safety of the two transgenic *Rehmannia glutinosa* lines. Agronomic traits and further safety evaluation shall be the focus in our further studies.

## 4. Materials and Methods

### 4.1. Reagents and Plant Material

Agar powder, MS medium, kanamycin, rifampicin, glufosinate ammonium (PPT), CTAB, Taq DNA polymerase, TransStart FastDNA polymerase, agarose biochemical reagent were purchased from Sigma (St. Louis, MO, USA). All primers were provided by Shanghai SANGON Biological Engineering Co., Ltd. (Shanghai, China) pCAMBIA3301 was digested with restriction endonuclease purchased from Shanghai Solarbio Bioscience & Technology Co., Ltd. (Shanghai, China).

Methanol and acetonitrile (HPLC Grade) were purchased from Fisher Scientific (Fair Lawn, NJ, USA). Distilled water, prepared by a Milli-Q water purification system (Millipore Corp., Bedford, MA, USA) was used throughout the study. Catalpol, aucubin, leonuride, acteoside and echinacoside were purchased from Beijing Qian He Biotechnology Co., Ltd. (Beijing, China).

*Rehmannia glutinosa* plants were obtained from Wuzhi County, Henan Province, China and authenticated by Professor Hongyan Liu (Henan Academy of Agriculture Sciences). The plants were maintained at 23–25 °C under 100 mmol/m^2^/s photosynthetic photon flux density with a day length of 16 h.

### 4.2. Construction of the Plant Expression Plasmid

The disarmed *A. tumefaciens* LBA4404 carrying the binary vector pCAM-TM35SCM was used to transform *Rehmannia glutinosa* plants. The plasmid was constructed as follows: the TMV-CP gene was amplified by PCR from the corresponding cDNA clone of TMV strain (*Rehmannia* mosaic virus, a member of the TMV), using primers with each appropriate restriction enzyme recognition site, and inserted into pCAMBIA3301 vector to form the pCAMBIA-TM vector. Then *HindIII-CaMV35S-GUS-Tnos-BstI* amplified using pBI121 vector as a template was inserted (pCAM-TM35S). The binary vector pCAM-TM35SCM was finally constructed by inserting CMV-CP gene which were cloned from the CMV Shandong isolate (SD-CMV) belonging to subgroup 1 with *SmaI* and *SacI* restriction sites into pCAM-TM35S.

### 4.3. Leaf Disc Transformation of Rehmannia glutinosa

Transgenic plants of *Rehmannia glutinosa* expressing the TMV-CP and CMV-CP genes were leaf-disc transformed with the *Agrobacterium tumefaciens* strain LB4404 containing the TMV-CP and CMV-CP genes. Infected explants were grown on MS medium with a feeder layer. After 2 days of culture, the explants were transferred onto callus medium prepared with 0.5 mg/mL claforan for the elimination of *Agrobacterium* cells. Medium must be replaced by fresh medium every 20 days until adventitious buds are differentiated. Thereafter, transgenic plants were selected on fresh MS plates that contained 0.5 mg/mL claforan and glufosinate (PPT). Regenerated shoots with PPT resistance were induced to root on one-half MS-medium containing 0.7% Phyto-agar.

### 4.4. PCR of Transgenic Plants

Genomic DNA of *Rehmannia glutinosa* was extracted from four-week-old plant leaves using a modified hexadecetyltrimethyl ammonium bromide (CTAB) method. About 0.2 g of fresh leaf tissue frozen in liquid nitrogen was ground in 1.5 mL Eppendorf tubes with a frozen mill. Then, 0.5 mL of 2% CTAB buffer was added into the tubes and incubated at 65 °C for 60 min. After incubation, 0.6 mL of chloroform–isoamyl alcohol (24:1, *v*/*v*) was added into the samples and mixed twice. The tubes were centrifuged for 10 min at 13,000 rpm under 4 °C. The supernatant was removed and 0.5 mL of isopropanol added and left at −20 °C for at least 3 days. The supernatant (0.5 mL) was transferred into a new tube and the DNA precipitated in 0.5 mL of 2-propanol at −20 °C for 2 h. The supernatant was removed after 10 min centrifugation at 13,000 rpm under 4 °C. The DNA was then washed with 70% ethanol and dissolved in TE buffer. The selected transformants were analyzed by PCR using TMV-CP (F-T1: 5’-ATGTCTTATACAATT GCAACTCCATCCCA-3’ and F-T2: 3’-TCAAGTTGCGGGACCAGAAGTCCAG-5’) and CMV-CP (R-C1: 5’-ATGTCTTATACAATTGCAACTCCATCCCA-3’ and R-C2: 3’-TCAAGTTGCGG GACCAGAAGTCCAG-5’) gene-specific primers.

### 4.5. RT-PCR of Transgenic Plants

The total leaf RNA (3 μg) was isolated from four-week-old plant leaves with TRIZOL reagent (Invitrogen Corp., Carlsbad, CA, USA) as described previously [31]. First-strain cDNA was synthesized using M-MLV Reverse Transcriptase (Promega Corp., Madison, WI, USA). For RT-PCR analysis, 1 μL of the first strand cDNA reaction products and high fidelity Ex-Taq polymerase (Takara Bio Inc., Kyoto, Japan) were used in a total volume of 30 μL. The reaction consisted of 27 cycles of 30 s at 95 °C, 40 s at 55 °C, and 1 min at 72 °C.

### 4.6. Southern Blotting

Southern-blotting analysis was carried out for confirming TMV-CP and CMV-CP genes were inserted into the chromosome of the transgenic strains. To generate templates for cDNA probes, the full-length TMV-CP and CMV-CP genes were cut out of plasmid pCAM-TM35SCM, respectively. cDNA Probes were labelled with digoxin by random primer method according to DIG High Prime DNA Labeling and Detection Starter Kit 1 (Roche Crop., Mannheim, Germany). Genomic DNA (10 μg) isolated from transgenic and wild-type *Rehmannia glutinosa* were digested with EcoRI, fractionated in a 0.7% agarose gel, and transferred to a nylon membrane. Hybridization and detection were performed according to the kit protocol.

### 4.7. TMV and CMV Infections

Before TMV and CMV infection, transgenic and wild-lines of *Rehmannia glutinosa* were transplanted into the greenhouse (20–28 °C, natural light) after seedling adaptation. Healthy plants were selected for the virus infection tests. A layer of quartz sand was evenly sprinkled on the first and second leaves at 2–3 leaf stage of the transplanted *Rehmannia glutinosa* seedlings. Then the leaves were rubbed gently using a brush with homologous TMV strain seeding solution prepared as described [32] and rinsed with distilled water. Control plants were inoculated using phosphate buffer solution without TMV. CMV (SD-CMV) infection operation followed the same procedure. The disease rate and disease index of the virus-invaded *Rehmannia glutinosa* was assessed according to grade and the investigation method of tobacco disease (Industry criteria of the tobacco industry of China, YC/T 39-1996).

### 4.8. Chromatographic Fingerprint Analysis of Rehmanniae Radix

The test samples were collected from the planting greenhouse and were dried at room temperature. Ten mg of each sample were weighed and extracted with ultrasound-assistance (32,000 Hz) for 5 min in 10 mL methanol at 25 °C. Methanol was used to make the final volume up to 10 mL. The samples were filtered through a 0.22 μm membrane before injection into the UPLC system. Separations were carried out with a Waters Acquity HSS T3 column (100 × 2.1 mm, 1.8 μm, Waters Corp., Milford, MA, USA). The mobile phase consisted of (A): water containing 0.1% formic acid; and (B): acetonitrile containing 0.1% formic acid; a gradient programmer was used according to the following profile: isocratic 2%B (0–1 min), linear gradient from 2% to 5% B (1–2 min), 5% to 12% B(2–5 min), 12% to 20% B (5–10 min), 20% to 30% B (10–12 min), 30% to 51.5% B (12–13.5 min), 50% to 100% B(13.5–16 min), isocratic 100% B for 2 min. The flow rate was 0.5 mL/min and column temperature was maintained at 30 °C. The injection volume was 1 μL. Mass spectrometry was performed with an Electron Spray Ionization (ESI) source operating in negative ion mode. The source temperature was set at 100 °C and the desolvation temperature was set at 450 °C with desolvation gas flow of 900 L/h. The capillary voltage and cone voltage were set to 3 kV and 40V, respectively. A mass range scanned was set from 50 to 1500 Da and Scan Time was 0.2 s^−1^.

Similarity Evaluation System for Chromatographic Fingerprints of TCMs recommend by the Chinese Pharmacopoeial committee was used for similarity evaluation of chromatographic patterns.

### 4.9. Determination of Leonurine, Echinacoside, Acteoside, Catalpol, Aucubin and Polysaccharides 

Simultaneous quantification of leonurine, echinacoside, acteoside, catalpol and aucubin was performed using a described HPLC-TQ-MS with SIR scan mode method [33]. Polysaccharides content were analyzed using a Solarbio Plant soluble sugar Kit (Beijing Solarbio Science & Technology Co., Ltd., Beijing, China). Numerical data were presented as mean ± standard deviation (S.D.). Data was analyzed using one-way ANOVA, followed by Student’s two-tailed t-test for comparison between wild and transgenic Rehmanniae Radix using the SPSS software package for Windows 11.5 (SPSS, Chicago, IL, USA).

## Figures and Tables

**Table 1:** The similarities of chromatograms of LBA-1 and LBA-2 to wild line.

Lines	1	2	3	4	5	6	7	8	9	10	Mean
LBA-1	0.899	0.941	0.893	0.916	0.918	0.989	0.99	0.987	0.991	0.982	0.951
LBA-2	0.940	0.953	0.899	0.910	0.902	0.937	0.926	0.928	0.991	0.987	0.937

**Table 2:** Contents of main compounds and polysaccharides in different gene type Rehmanniae Radix.

Lines	Polysaccharides (mg/g)	Main Compounds (mg/g)				
		Aucubin	Catalpol	Ajugol	Acteoside	Echinacoside
Wild lines	33.66 ± 1.82	17.80 ± 2.88	683.57 ± 120.12	189.21 ± 22.49	1.25 ± 0.45	5.71 ± 1.34
LBA-1	33.47 ± 1.61	17.84 ± 3.55	687.20 ± 112.35	186.93 ± 18.11	1.25 ± 0.46	5.74 ± 2.34
LBA-2	33.22 ± 1.69	17.76 ± 3.75	682.67 ± 132.77	187.59 ± 22.12	1.26 ± 0.28	5.73 ± 1.60

## Supplementary Materials

Supplementary materials can be accessed at: http://www.mdpi.com/1420-3049/21/9/1134/s1.
