# Supplementary material for: Construction and Quality Analysis of Transgenic Rehmannia glutinosa Containing TMV and CMV Coat Protein
Source: Molecules. 2016 Aug 27;21(9):1134. doi: 10.3390/molecules21091134 (PMC6273745; doi:10.3390/molecules21091134)
Supplement: Supplementary file 1 [file molecules-21-01134-s001.pdf]

Supplementary Materials: Construction and Quality Analysis of Transgenic *Rehmannia glutinosa* Containing TMV and CMV Coat Protein

Zhongqiu Teng, Ye Shen, Jing Li, Zhongping Lin , Min Chen, Min Wang, Man Li, Hongran Dong and Luqi Huang

Table S1. L16 (4^5) Factors and levels in orthogonal test.

Levels	Factors				
	A (h)	B (h)	C (min)	D (OD)	E (mg/L)
1	0	24	4	0.2	2+0.5
2	24	36	6	0.4	2+0.2
3	36	48	8	0.6	4+0.5
4	48	60	10	0.8	4+0.2

A: pre-culture time (h), B: co-culture time (h), C: Infection time (min), D: bacteria concentration (OD), E: phytohormones 6-BA+NAA (mg/L).

Table S2. Results .of L16 (4^5) orthogonal test.

N.O.	A (h)	B (h)	C (min)	D (OD)	E (mg/L)	Bud Ratio	Positive Rate
1	1	1	1	1	1	0.334	0
2	1	2	2	2	2	0.464	0.071
3	1	3	3	3	3	0.278	0.063
4	1	4	4	4	4	0	0
5	2	1	2	3	4	0.084	0.111
6	2	2	1	4	3	0.056	0
7	2	3	4	1	2	0.25	0.077
8	2	4	3	2	1	0.292	0.286
9	3	1	3	4	2	0.042	0.5
10	3	2	4	3	1	0	0
11	3	3	1	2	4	0.209	0.125
12	3	4	2	1	3	0.229	0
13	4	1	4	2	3	0.028	1
14	4	2	3	1	4	0.389	0
15	4	3	2	4	1	0.042	0.5
16	4	4	1	3	2	0.334	0.154
K1	0.269	0.122	0.233	0.300	0.167		
K2	0.170	0.227	0.205	0.248	0.273		
K3	0.120	0.195	0.250	0.174	0.148		
K4	0.198	0.214	0.070	0.035	0.170		
R	0.149	0.105	0.180	0.265	0.125		
K1	0.034	0.403	0.070	0.019	0.197		
K2	0.118	0.018	0.017	0.370	0.201		
K3	0.156	0.191	0.212	0.082	0.266		
K4	0.413	0.110	0.269	0.250	0.059		
R	0.379	0.385	0.199	0.351	0.207		

A: pre-culture time (h), B: co-culture time (h), C: Infection time (min), D: bacteria concentration (OD), E: phytohormones 6-BA+NAA (mg/L).

Table S3. Variance analysis of orthogonal experiment.

Factor	Dev Sq	Degree of Freedom	F Factor	P Factor
A	0.046	3	1.769	>0.05
B	0.026	3	1.000	>0.05
C	0.081	3	3.115	>0.05
D	0.160	3	6.154	>0.05
E	0.038	3	1.462	>0.05
Deviation	0.030	3		

$F_{0.05\ (3,\ 3)} = 9.28$; A: pre-culture time (h), B: co-culture time (h), C: Infection time (min), D: bacteria concentration (OD), E: phytohormones 6-BA+NAA (mg/L).

Table S4. Variance analysis of orthogonal experiment.

Factor	Dev Sq	Degree of Freedom	F Factor	P Factor
A	0.321	3	3.776	> 0.05
B	0.324	3	3.812	> 0.05
C	0.085	3	1.000	> 0.05
D	0.307	3	3.612	> 0.05
E	0.091	3	1.071	> 0.05
Deviation	0.090	3		

$F_{0.05\ (3,\ 3)} = 9.28$; A: pre-culture time (h), B: co-culture time (h), C: Infection time (min), D: bacteria concentration (OD), E: phytohormones 6-BA+NAA (mg/L).

Table S5. Inoculation concentration test of TMV to *Rehmannia glutinosa* plants.

Inoculation Time (Day)	Inoculation Concentration (μg/mL)				
	0.07	0.10	0.50	1.00	10.00
2	0	0	0	0	100%
4	0	40%	80%	100%	100%
8	0	60%	100%	100%	100%
10	20%	60%	100%	100%	100%
14	20%	60%	100%	100%	100%

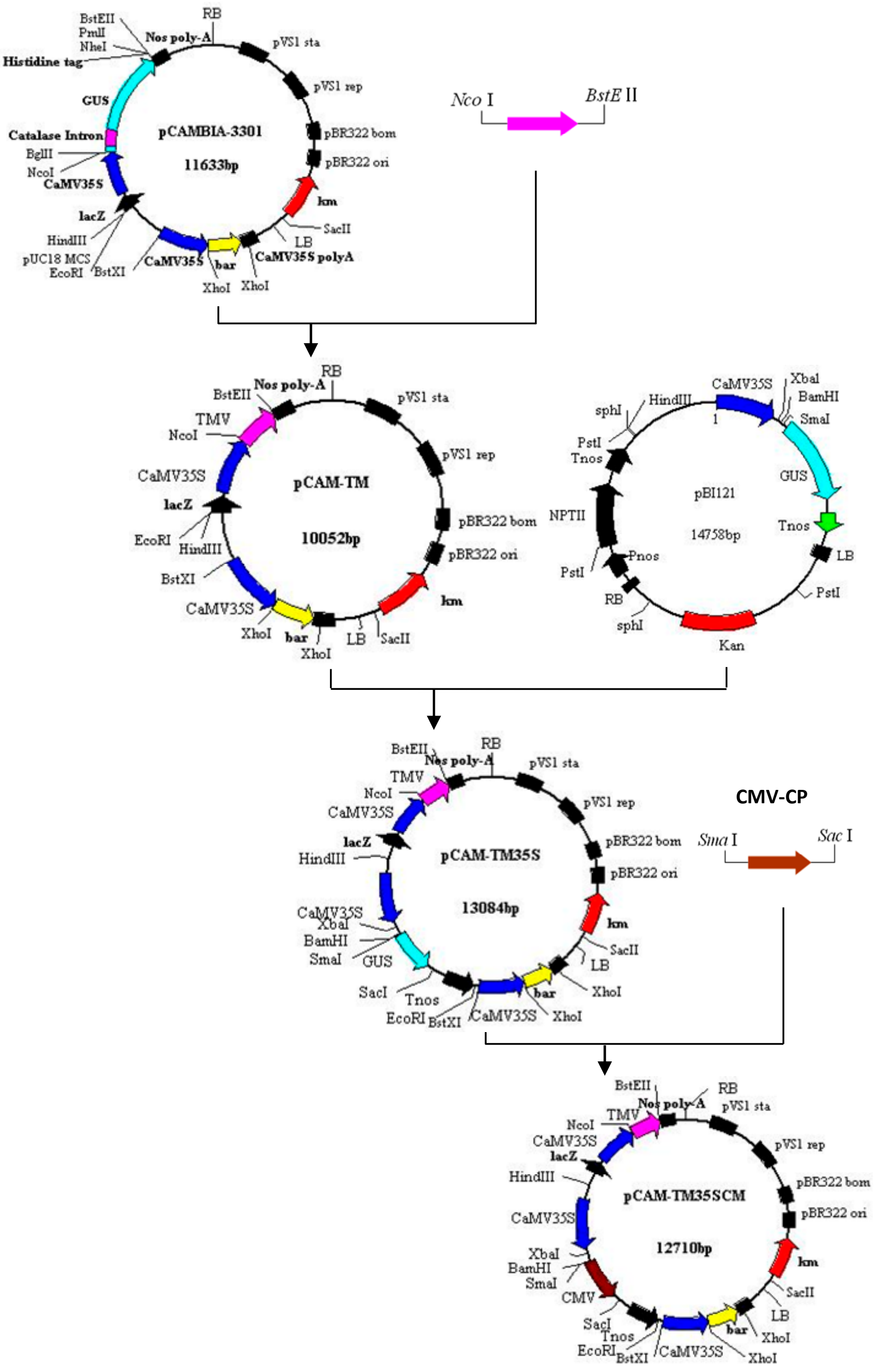

Figure S1. Construction of the plant expression plasmid.

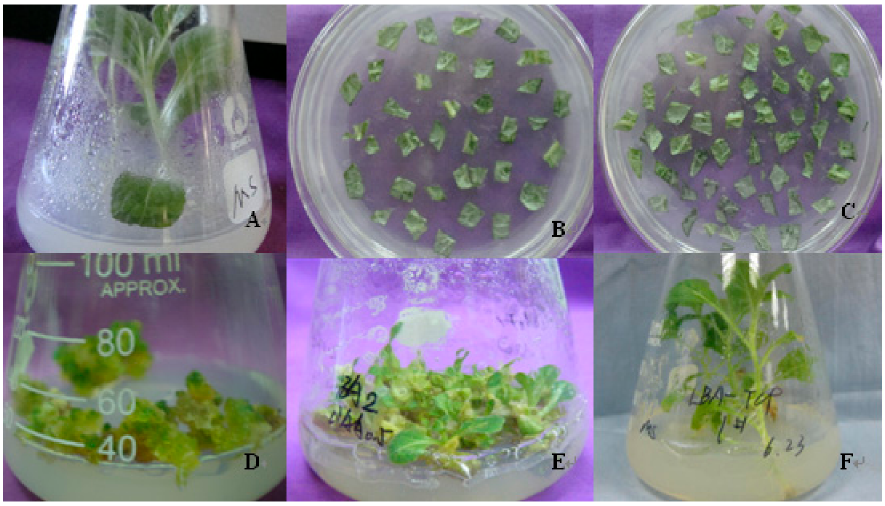

Figure S2. The growth process of the *Rehmannia* from transformed leaf discs. **A**. plantlets; **B**. pre-culture; **C**. co-culture; **D**. resistant callus differentiation; **E**. resistant bud differentiation; **F**. regenerated plantlets by PPT screening.

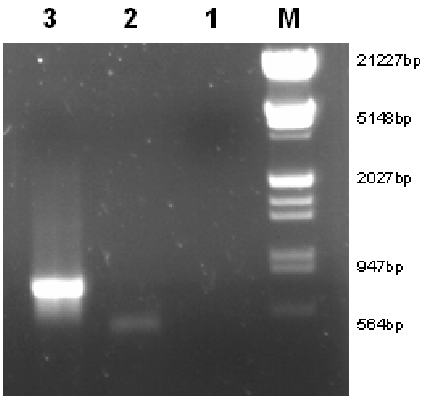

Figure 3. Probe amplified by PCR. M: Marker; 1: Control (ddH_2O); 2: PCR product of TMV probe; 3: PCR product of CMV probe.

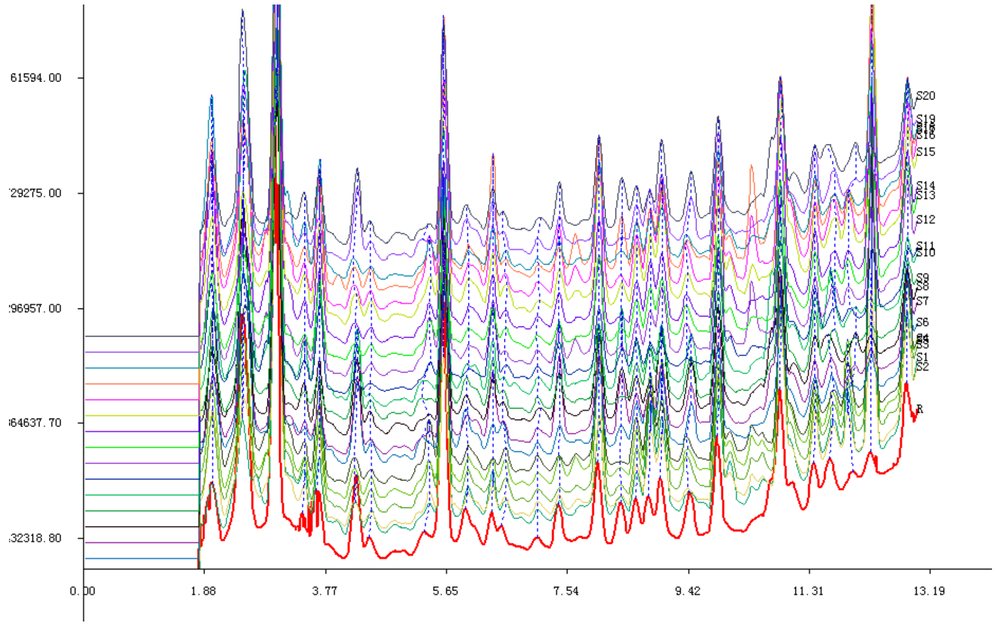

(**a**)

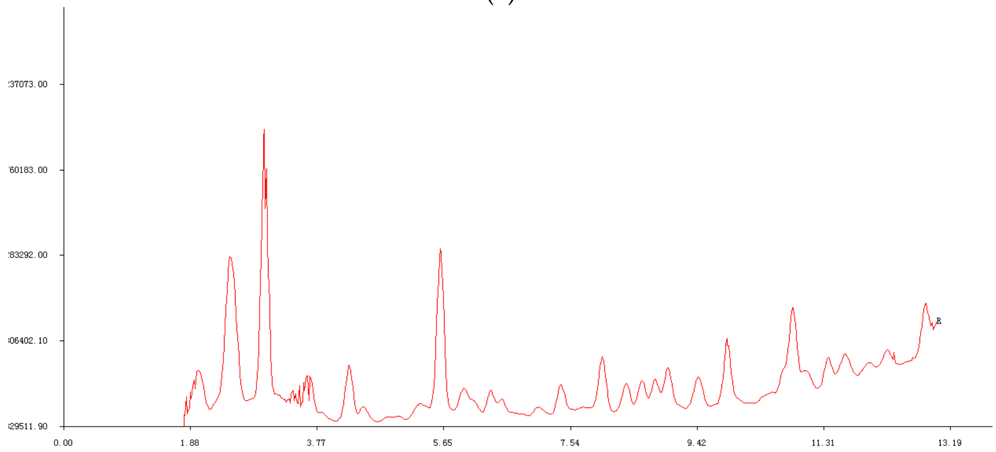

(**b**)

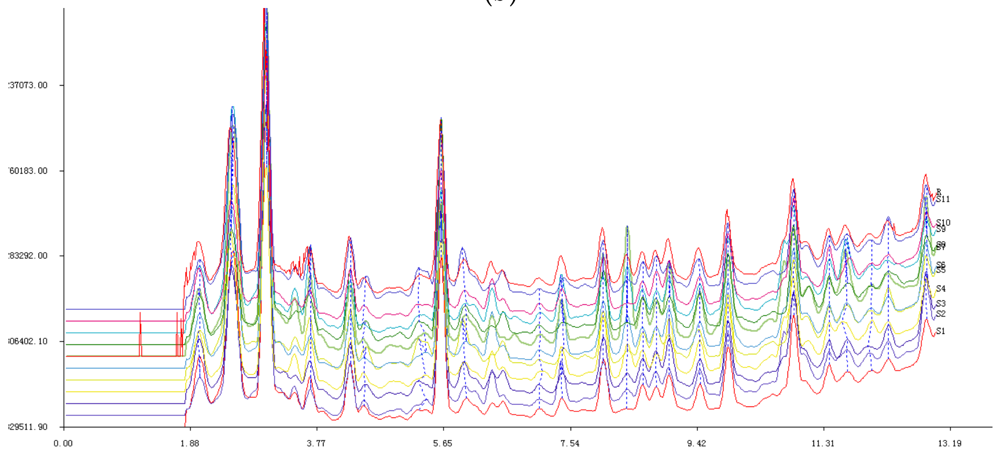

(**c**)

Figure S4. Chromatographic Fingerprint analysis of Rehmanniae Radix. (**a**) Chromatographic Fingerprint of wild lines; (**b**) Reference Chromatograph; (**c**) Comparation of Transgenic *Rehmannia glutinosa* to reference chromatopraph.
